# Supplementary material for: Improving gene function predictions using independent transcriptional components
Source: Nat Commun. 2021 Mar 5;12:1464. doi: 10.1038/s41467-021-21671-w (PMC7935959; doi:10.1038/s41467-021-21671-w)
Supplement: Supplementary file 1 — Supplementary Information [file 41467_2021_21671_MOESM1_ESM.pdf]

SUPPLEMENTARY FIGURES

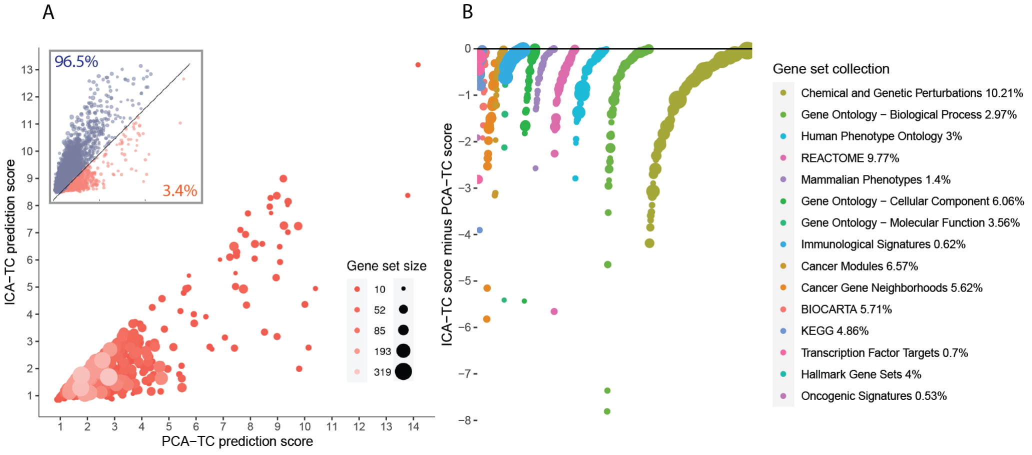

Supplementary Figure 1: Gene sets where the PCA–TC based method provided better median prediction scores. **A)** Scatterplot of PCA-TC (x-axis) versus ICA-TC (y-axis) based median prediction scores. The scatterplot only shows the 811/23,413 (3.4%) of gene sets for which the PCA-TC based method produced better median prediction scores in comparison to the ICA-TC based method. The size and color of dots represent the number of genes in a gene set. The inset shows the scatterplot with all 23,413 gene sets. **B)** Difference between the PCA-TC and ICA-TC based median prediction scores (y-axis) for the 811 gene sets that show higher prediction scores for the PCA-TC based method. The gene sets are ranked on the x-axis based on the difference and grouped according to gene set collections. The legend shows each gene set collection and the percentage of gene sets from that collection that showed better median prediction scores with the PCA-TC based method

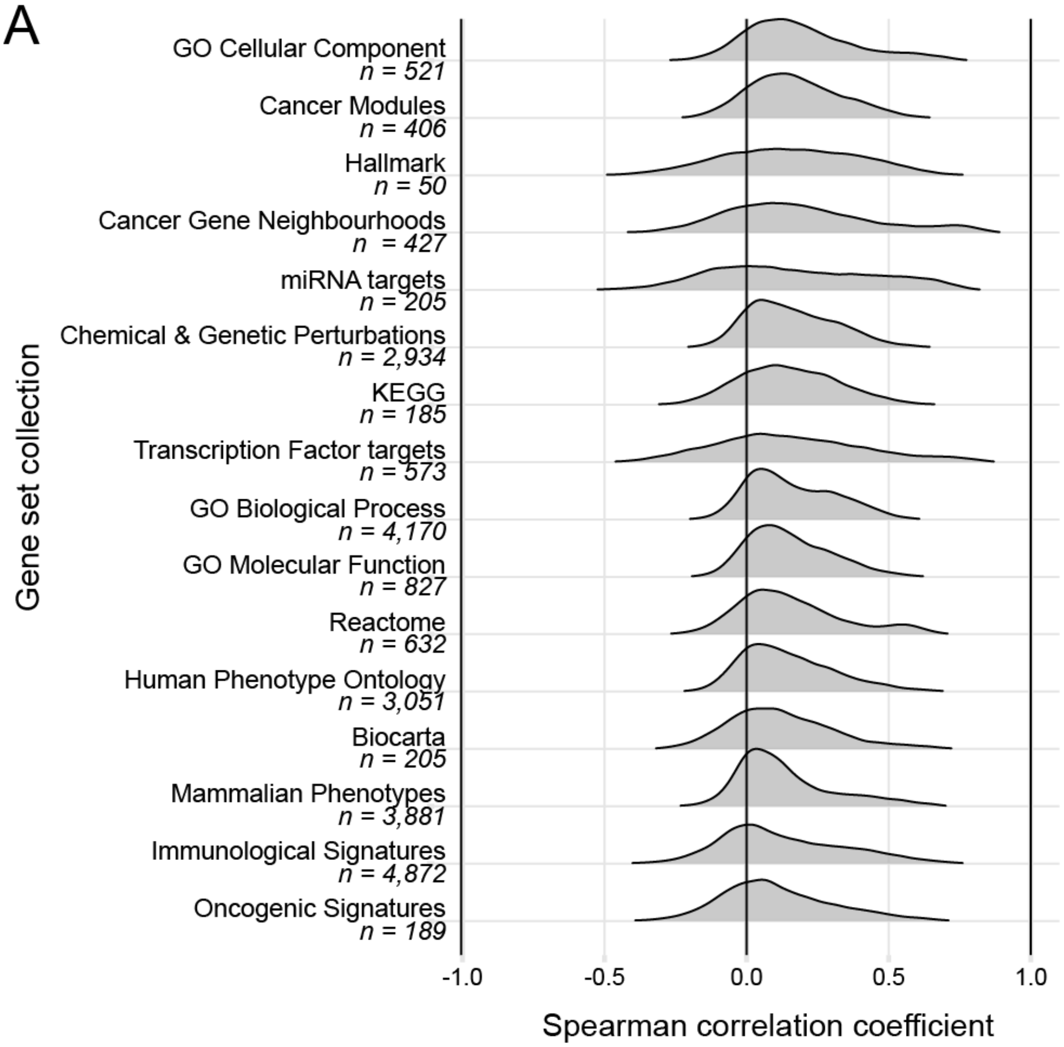

Supplementary Figure 2: Cross-species prediction score correlations. Histograms showing the distributions of Spearman correlation coefficients between the ICA-TC based prediction scores of every mouse-human gene ortholog pair (n = 14,589) for 16 gene set collections. Median Spearman correlation between ortholog prediction scores ranged between 0 and 0.2 for every gene set collection. The number of gene set perspective comparisons performed for each collection is depicted in the y axis text.

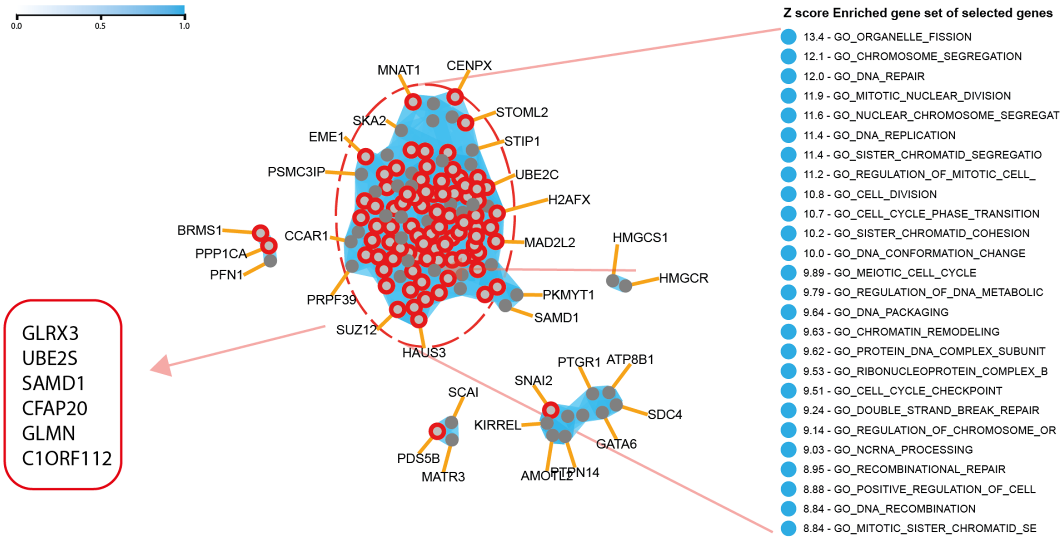

Supplementary Figure 3: Co–functionality network using essential genes for survival upon treatment with CD437. The highlighted cluster contains 114 genes that are (red) or are not (grey) members of a DNA repair gene set. This entire cluster of 114 genes is enriched for gene sets that are indirectly related to the cellular response to DNA damage (depicted on right) such as cell cycle, chromosome segregation together with the DNA repair gene set (Z–score 12). A selected group of genes that are still underexplored in the context of the cellular response to DNA repair is depicted on the left. The cluster depicts as edges all pairs of genes with an absolute Pearson correlation bigger than 0.7. Genes with no edges above the threshold are not depicted.

SUPPLEMENTARY NOTES

Consensus independent component analysis (c-ICA)

Gene expression profiling for the samples included in this study was performed with complex tumor biopsies. These biopsies contain a complex mixture of heterogeneous tumor cells and non-tumor cells present in the tumor microenvironment. The resulting profiles represent the average expression patterns of cells present in the biopsies. Consensus ICA was utilized to segregate the average expression patterns of complex biopsies into statistically independent transcriptomic footprints [1]. Applying ICA on a mRNA expression dataset with p genes and n samples results in the extraction of i independent components of dimension $1xp$ (hereafter called estimated sources, ESs) and a mixing matrix of dimension ixn which contains the coefficients of ESs in each sample. The weight of each ES represents the direction and magnitude of its effect on the expression level of each gene and the coefficients of MM represent activity scores of the ESs in the corresponding sample. In ICA, a pre–processing technique called whitening is applied on the input dataset to make the estimation more time efficient. Whitening was used to transform gene expression profiles of all samples so that the transformed profiles are uncorrelated and have variance of one. Next, ICA was performed on the whitened dataset using the FastICA function from the FastICA package (version 1.2.0), resulting in the extraction of i independent components and a mixing matrix. The parameter i was chosen as the number of top principal components which captured 90% of the total variance seen in the whitened dataset. In ICA, an initial random weight vector with a variance of 1 has to be chosen in order to obtain statistically independent ESs. Hence, varying initial random weight vectors could result in different sets of ESs. To retrieve a set of consensus ESs (or CESs), we performed 25 ICA runs, each with a different random initialization weight vector. The assumption of above method to obtain CESs is that over a large number of runs of ICA, FastICA algorithm does not converge to any local solution for most of the runs. The ESs extracted from these runs were clustered together if absolute value of Pearson correlation between them was > 0.9. Clusters with sources in $> 50\%$ of the runs were used to obtain CESs.

Guilt–by–association procedure to generate gene predictions

The procedure was done for each gene set collection separately and used as input the weights from the mixing matrix. Two groups of genes were defined for each specific gene set in a collection: The IN group is composed of all genes assigned to that gene set, the OUT group is composed of all genes not assigned to that gene set. We generated a vector of means using the algebraic average of the rows of the mixing matrix corresponding to genes of the IN group. Next, for every candidate gene in the OUT group we performed an association test using Distance correlation between the vector of means and the row of the candidate gene in question. To generate predictions for genes already in the IN group a modified procedure was followed. For every each gene in the IN group we regenerated a new vector of means using only the algebraic average of all other genes in the IN group. Next, we performed the same association test between the row corresponding to that gene and the regenerated vector of means. For every gene in the IN or OUT groups a thousand random association test were performed on vectors of means generated by taking a random sample of genes from the mixing matrix of the same size as the number of members of the gene set in question. A kernel density estimator was fitted to the resulting distribution. The procedure results in a matrix (gxs) where g is the number of genes and s is the number of gene sets. Each column of this matrix represents the probability of each gene belonging to a gene set. Each row represents a functionality barcode for each gene.

Comparison between the prediction scores of ICA–TCs and PCA–TCs

The comparison procedure was done for each gene set collection separately and for ICA-TC and PCA–TC prediction scores separately. For each specific gene set predictions we define two groups of genes: the IN group is composed of all genes assigned to that gene set, the OUT group is composed of all genes not assigned to any gene set in that collection. A vector of means using the algebraic average was calculated per gene set using the prediction scores corresponding to genes in the IN group and another one using prediction scores of the

OUT group. We performed a Mann-Whitney U test per gene set collection between PCA-IN medians and ICA-IN medians and between PCA-OUT medians and ICA-OUT medians corresponding to the gene sets in that gene set collection.

Evaluating the predictive power of ICA-TCs and PCA-TCs using old gene annotations

Old versions of the MSigDB C5 subset where downloaded from the MSigDB archive (version 3.0). A prefix "GO_" was added to all gene set names and all identical matches with the gene set names of version 6.2 were retained for analysis. In these matching gene sets, only gene-sets that increased in their memberships in between versions were retained for further analysis. Genes that changed in between versions are defined as 'update genes'. Prediction scores were separately calculated using version 3.0 and version 6.2 of the C5 MSigDB subset. Median prediction scores were calculated for each gene set using only updated genes in both version 3.0 and version 6.2 generated prediction scores. For each of the gene set collections we performed Mann-Whitney U test between the median prediction scores of version 3.0 and version 6.2. This entire procedure was performed once using ICA-TC and once suing PCA-TC based predictions.

Association with multifunctionality

The comparison procedure was done for each gene set database separately and for ICA and PCA predictions separately. We calculated the multifunctionality score of each gene as described in the original publication [2]. Again we calculated the distance correlation between the vector composed of the prediction scores of all genes for a specific gene set and the vector of the corresponding multifunctionality scores for each gene. We performed Mann-Whitney U test between correlation statistics obtained with ICA-TCs and obtained with PCA-TCs.

Acquiring mouse mRNA expression profiles

We downloaded mRNA expression profiles of all mouse samples available for the Affymetrix Mouse Genome 430 2.0 microarray platform in their unprocessed CEL format (GPL1261). To remove potential duplicates MD5 digests were calculated for all CEL files and one replicate was retained. All CEL files were then transformed to gene–level normalized expression values using the RMAExpress algorithm (1.20.0). To remove potential bad quality experiments principal component analysis (PCA) was applied to the normalized expression matrix. In microarray expression data principal component one (PC1) captures a platform–specific signature that is shared by all samples. Samples that do not share this signature may represent bad quality experiments. All samples with a PC1 lower than the 80th percentile were dropped ending up with 56,657 samples.

Using the same methodology used for the human microarray we subsequently applied PCA again to whiten the normalized expression matrix and observed that the number of components needed to capture 90% of the dataset variance was 753. We then applied independent component analysis to the whitened matrix targetting the acquisition of 753 components on 25 different runs. We retained sources present in at least 13 runs (Credibility index = 0.5) ending up with 614 consensus mouse ICA-TCs. As multiple probesets can target a single gene on Affymetrix gene expression microarrays we utilized The R package Jetset (version 3.4.0) to obtain one–to–one mapping between genes and the highest quality probesets for expression data generated with the Affymetrix Mouse Genome 430 2.0 microarray platform [3].

Creating mouse gene set collections based on Molecular Signal Database human gene set collections

To generate gene sets in gmt-file format based on the human gene set collections from Molecular Signatues Database v6.2, human gene entrez IDs were translated to mouse gene entrez IDs using the annotations provided in the msigdbr R package (version 7.2.1). The msigdbr package was built manually using the "msigdbr-prepare.R script", that was available at

https://github.com/igordot/msigdbr/, to be able to collect Molecular Signatues Database v6.2 gene sets: line 14 was modified (msigdb version = 6.2). For Human Phenotype Ontology and Mammalian Phenotypes gene set collections human entrez IDs were translated to mouse entrez IDs by using the BioMart datasets (ensembl) available through the biomaRt R package (version 2.45.9).

Predictable co-expression patterns

Using the 'hclust' function we performed a hierarchical clustering using the 'ward.D2' method over an input distance matrix generated from the c-ICA mixing matrix using distance correlation ($1 - correlation$). A cutoff of 2.5 was selected because it created clusters of sizes within the range of 10-500 which is the range used for gene sets. This resulted in 173 clusters of sizes ranging from 13 to 389 with a mean of 115. The density of a cluster was defined as the median correlation value when subsetting the distance matrix for that cluster. Uncharacterized genes were defined as genes with the 'C*orf*' or the 'LOC*' glob pattern in their gene symbol. Using all 16 prediction score matrices the maximum prediction score per gene was selected. A median of this maximum prediction score was generated per cluster and correspond to the cluster predictability. A Pearson correlation coefficient was calculated between the cluster densities and median maximum prediction scores using 'cor.test'.

Data acquisition RNA-seq samples

We downloaded and quality controlled the sample subset processed in the manuscript by Deelen et al using the ftp link table provided in the Supplementary Notes of the original publication [4]. We generated pseudocounts using Kallisto 0.46.0 [5] specifying default parameters for paired-end data and the following additional settings for single-end data: –single -l 200 -s 20 -bias. The following two genome files were merged and used to create the Kallisto index:

`ftp://ftp.ensembl.org/pub/release-101/fasta/homo_sapiens/cdna/Homo_sapiens.GRCh38.cdna.all.fa.gz`

`ftp://ftp.ensembl.org/pub/release-101/fasta/homo_sapiens/ncrna/Homo_sapiens.GRCh38.ncrna.fa.gz`

In total 31,395 samples were successfully downloaded. For quality control, we dropped samples with less than 70% of pseudoaligned reads and where the read count did not match the count reported by the ftp link table, leaving us with 30,288 samples. For duplicated samples with a correlation of > 0.9999, one was randomly selected and the others removed which left 29,138 samples for our analysis. Next, transcripts that were not located on the main chromosomes (1-22, X, Y, MT), but on scaffolds were removed, reducing the number of transcripts from 250,156 to 228,267. Another 965 transcripts with 100% identical sequences were removed to avoid a double-mapping bias while randomly keeping one transcript. Transcripts with less than one non zero counts were dropped, ending up with 227,300 transcripts.

The transcript counts per sample were summed up to gene-level counts for each sample, which yielded 59,030 genes. Duplicated gene names ocurred in 14 instances and were removed while keeping the newest version of each. At last, we removed genes expressed in less than 1% of the samples, ending up with a final gene-count matrix of 58,433 genes and 29,138 samples. Finally, the gene counts were normalized using size factors and variance stabilizing transformation from DESeq2 1.26.0 [6].

Comaprison of RNA-seq and microarray based predictions

To compare predictions obtained between RNA-seq and microarray datasets, Ensembl gene IDs were mapped to entrez IDs. A table containing both was downloaded from HGNC (https://pubmed.ncbi.nlm.nih.gov/30304474/) on 19.10.2020. Missing NCBI IDs were filled with the newest non-curated ones, supplied directly by NCBI, ending up with 38,381 genes identified with Ensembl gene IDs to entrez IDs mapping.

References

1 Chiappetta, P., Roubaud, M. C. & Torrésani, B. Blind source separation and the analysis of microarray data. *J. Comput. Biol.* **11**, 1090–1109 (2004).

2 Gillis, J. & Pavlidis, P. The impact of multifunctional genes on "guilt by association" analysis. *PLoS ONE* **6**, e17258 (2011).

3 Li, Q., Birkbak, N. J., Gyorffy, B., Szallasi, Z. & Eklund, A. C. Jetset: selecting the optimal microarray probe set to represent a gene. *BMC Bioinformatics* **12**, 474 (2011).

4 Deelen, P. *et al.* Improving the diagnostic yield of exome- sequencing by predicting gene–phenotype associations using large-scale gene expression analysis. *Nat Commun* **10**, 1–13 (2019).

5 Bray, N. L., Pimentel, H., Melsted, P. & Pachter, L. Near-optimal probabilistic RNA-seq quantification. *Nat Biotechnol* **34**, 525–27 (2016).

6 Love, M. I., Huber, W. & Anders, S. Moderated estimation of fold change and dispersion for RNA-seq data with DESeq2. *Genome Biol* **15**, 550 (2014).
