# Supplementary material for: Improving gene function predictions using independent transcriptional components
Source: Nat Commun. 2021 Mar 5;12:1464. doi: 10.1038/s41467-021-21671-w (PMC7935959; doi:10.1038/s41467-021-21671-w)
Supplement: Supplementary file 3 — Description of Additional Supplementary Files [file 41467_2021_21671_MOESM3_ESM.pdf]

Description of Additional Supplementary Files

File Name: Supplementary Data 1
Description: Mean prediction scores for gene sets in 16 gene set collections.

File Name: Supplementary Data 2
Description: Gene-wise multifunctionality associations for 16 Gene set collections.

File Name: Supplementary Data 3
Description: Gene clusters derived from the mixing matrix of human microarray ICA.

File Name: Supplementary Data 4
Description: Literature evidence for the predicted functionality of uncharacterized genes.
