# Supplementary material for: Improving gene function predictions using independent transcriptional components
Source: Nat Commun. 2021 Mar 5;12:1464. doi: 10.1038/s41467-021-21671-w (PMC7935959; doi:10.1038/s41467-021-21671-w)
Supplement: Supplementary file 8 — Reporting Summary [file 41467_2021_21671_MOESM8_ESM.pdf]

nature research

Corresponding author(s): R.S.N. Fehrmann

Last updated by author(s): Jan 11, 2021

Reporting Summary

Nature Research wishes to improve the reproducibility of the work that we publish. This form provides structure for consistency and transparency in reporting. For further information on Nature Research policies, see our Editorial Policies and the Editorial Policy Checklist.

Statistics

For all statistical analyses, confirm that the following items are present in the figure legend, table legend, main text, or Methods section.

n/a	Confirmed	
☐	☒	The exact sample size (n) for each experimental group/condition, given as a discrete number and unit of measurement
☒	☐	A statement on whether measurements were taken from distinct samples or whether the same sample was measured repeatedly
☐	☒	The statistical test(s) used AND whether they are one- or two-sided<br>*Only common tests should be described solely by name; describe more complex techniques in the Methods section.*
☒	☐	A description of all covariates tested
☐	☒	A description of any assumptions or corrections, such as tests of normality and adjustment for multiple comparisons
☐	☒	A full description of the statistical parameters including central tendency (e.g. means) or other basic estimates (e.g. regression coefficient) AND variation (e.g. standard deviation) or associated estimates of uncertainty (e.g. confidence intervals)
☐	☒	For null hypothesis testing, the test statistic (e.g. F, t, r) with confidence intervals, effect sizes, degrees of freedom and P value noted<br>*Give P values as exact values whenever suitable.*
☒	☐	For Bayesian analysis, information on the choice of priors and Markov chain Monte Carlo settings
☒	☐	For hierarchical and complex designs, identification of the appropriate level for tests and full reporting of outcomes
☐	☒	Estimates of effect sizes (e.g. Cohen's d, Pearson's r), indicating how they were calculated

Our web collection on statistics for biologists contains articles on many of the points above.

Software and code

Policy information about availability of computer code

Data collection	No software was used for data collection.
Data analysis	ICA-analysis and creating the network was performed using Analyzertool5 (in-house developed, available through https://bitbucket.org/groupfehrmann/analyzertool/src/master/). [Analysis>Component Analysis>Independent Component Analysis] was used for ICA, [Tools>Create gene network] was used for generating prediction scores.<br><br>Aggregation of raw expression data was performed according to the robust multi–array average algorithm with RMAExpress (version 1.1.0).<br><br>Within R (version 3.4.3), hierarchical clustering was performed using the hclust() function ('stats' package version 3.4.3); the package ‘jetset’ (version 3.4.0) was used to obtain one–to–one mapping between genes and the most representative probe sets on the Affymetrix HG–U133 Plus 2.0 platform; the gplots (version 3.1.0) and ggplot2 (version 3.3.2) packages were used to generate heatmaps and plots, respectively. The msigdbr R package (version 6.2) was used to obtain mouse gene set collections; for Human Phenotype Ontology and Mammalian Phenotypes gene set collections human entrez IDs were translated to mouse entrez IDs by using the BioMart datasets (ensembl) available through the biomaRt R package (version 2.45.9).

For manuscripts utilizing custom algorithms or software that are central to the research but not yet described in published literature, software must be made available to editors and reviewers. We strongly encourage code deposition in a community repository (e.g. GitHub). See the Nature Research guidelines for submitting code & software for further information.

Data

Policy information about availability of data

All manuscripts must include a data availability statement. This statement should provide the following information, where applicable:

- Accession codes, unique identifiers, or web links for publicly available datasets
- A list of figures that have associated raw data
- A description of any restrictions on data availability

Prediction score tables, and corresponding input matrices for ICA and PCA based prediction scores for all 16 gene set collections are available on the download section of www.genetica-network.com
Data associated with the main figures and mouse-human prediction score correlations are available at Figshare:
https://doi.org/10.6084/m9.figshare.13265159

Field-specific reporting

Please select the one below that is the best fit for your research. If you are not sure, read the appropriate sections before making your selection.

☒ Life sciences ☐ Behavioural & social sciences ☐ Ecological, evolutionary & environmental sciences

For a reference copy of the document with all sections, see nature.com/documents/nr-reporting-summary-flat.pdf

Life sciences study design

All studies must disclose on these points even when the disclosure is negative.

Sample size	We obtained publicly available data for three different platforms; GPL570 (n=106,462), RNA-seq (n=29,138), GPL1261 (n=25,585). All available samples were collected to maximize the power of the analysis. To investigate the samples, download the 'mixing matrix' for each platform at the 'support data' tab at www.genetica-network.com.
Data exclusions	Quality control of expression profiles ("samples") was performed as follows: 1) we removed profiles of technically bad quality; 2) in GEO it is allowed to reupload profiles; to obtain a dataset with unique profiles, we opted to remove duplicates based on identical MD5 digests; 3) in the microarray dataset, many probes can map the same gene, therefore to obtain unique measurements for each gene, low-quality probes were removed following the jetset-methodology (R-package 'jetset' v3.4.0); 4) in the case of RNA-seq profiles, genes with identical sequences cannot be distinguished by the pseudo-counter, and were therefore removed; 5) We removed genes that were expressed in less than 5% of our RNA-seq dataset.<br>In total, this meant that 54,895 samples were excluded from the GPL570 platform dataset, 31,288 samples from the GPL1271 platform dataset, and 2,257 samples for the RNA-seq dataset.
Replication	To ensure to acquire robust sources, we generate consensus sources from 25 different ICA-runs.
Randomization	Randomization was not necessary, since our analysis was not prospective. Our method prioritizes genes, but it is not feasible to evaluate the predictive power of the predictions prospectively.
Blinding	Gene set predictions of member genes were calculated assuming they were not members of the gene set they belong to.

Reporting for specific materials, systems and methods

We require information from authors about some types of materials, experimental systems and methods used in many studies. Here, indicate whether each material, system or method listed is relevant to your study. If you are not sure if a list item applies to your research, read the appropriate section before selecting a response.

Materials & experimental systems

n/a	Involved in the study	
☒	☐	Antibodies
☒	☐	Eukaryotic cell lines
☒	☐	Palaeontology and archaeology
☒	☐	Animals and other organisms
☒	☐	Human research participants
☒	☐	Clinical data
☒	☐	Dual use research of concern

Methods

n/a	Involved in the study	
☒	☐	ChIP-seq
☒	☐	Flow cytometry
☒	☐	MRI-based neuroimaging
